# Antioxidant effect of different vitamins on methemoglobin production: An *in vitro* study

**Published:** 2012

**Authors:** Nahid Atyabi, Seyedeh Parastoo Yasini, Seyedeh Missagh Jalali, Hamid Shaygan

**Affiliations:** 1 *Department of Clinical Pathology, Faculty of Veterinary Medicine, University of Tehran, Tehran, Iran; *; 2 *Faculty of Veterinary Medicine, Azad Islamic University, Garmsar branch, Garmsar, Iran.*

**Keywords:** Vitamin, Methemoglobin, Antioxidant, Nitrite, *in vitro*

## Abstract

Nitrite intoxication occurs frequently in ruminants and equines. The most common treatment of this disorder is administration of 1% methylene blue, although the use of some antioxidant agents e.g. vitamins and complementary treatment may also be useful. The aim of this study was to evaluate the *in vitro* antioxidative effects of some vitamins on methemoglobinemia induced by sodium nitrite. For this purpose the blood sample of a healthy dairy cattle was pre-incubated with three different concentrations (5, 10, 20 mmol L^-1^) of each vitamin (E, C, B1, A and a combination of vitamin E and vitamin C) as antioxidant agent at 4 ^°^C for 24 hours. A control group with normal saline instead of vitamin was applied. Then, all samples were treated with sodium nitrite (10 mmol L^-1^) as an oxidant agent for 10 minutes and the level of methemoglobin formation was measured spectrophoto-metrically. The results revealed that the level of methemoglobin decreased significantly (*P *< 0.05), when vitamin E (10 and 20 mmol L^-1^) and vitamin C (5 mmol L^-1^) was applied to the tests, separately. Vitamin C at the concentration of 20 mmol L^-1^, was not effective, but it even increased methemoglobin formation significantly. Combination of vitamin E and C was significantly effective at concentration 5 mmol L^-1^, but not at concentration 10 and 20 mmol L^-1^. Vitamin A and vitamin B_1 _were not effective in any concentration. It was concluded that vitamins especially vitamin C and E can reduce oxidative effects which induced methemoglobin formation *in vitro* and could be used as an alternative medication.

## Introduction

Oxidative stress of erythrocytes can cause destruction of iron complex and hemoglobin products formation.^1^ When the erythrocyte antioxidant defenses are overloaded, hemolysis can occur due to the inability of the erythrocytes to regenerate the affected components.^2^ Hereditary or acquired methemoglobinemia, is a clinical condition in which the hemoglobin is oxidized to methemoglobin that contains oxidized ferric iron Fe^+3^ rather than the reduced ferrous form Fe^+2^ found in hemoglobin. Ferric iron has slightly greater affinity for oxygen which shifts the oxygen dissociation curve to the left resulting in decreased release of oxygen in tissues.^3^^,^^4^

Nitrite is an oxidative agent that causes methemoglobinemia in many species. Ruminants are especially vulnerable because the ruminal flora reduces nitrate to ammonia, while nitrite as an intermediate product is 10 times more toxic than nitrate. Acute intoxication is manifested primarily by methemoglobin formation and resultant anoxia.^5^ The common treatment of methemoglobinemia with methylene blue is potentially hazardous and should not be used specially in patients who may be at risk or suffer from glucose-6-phosphate dehydrogenase (G6PD) deficiency.^6^ Therefore, the other ways of treatment are suggested for this condition, such as using various vitamins as antioxidant.^7^

Vitamin C has potentials to scavenge free radicals and protect cells from oxidative damage. Recycling of α-tocopherol by ascorbate has been demonstrated in cellular organelles and erythrocyte membranes.^8^^-^^11^ It also acts as a co-factor for NADP reductase required for glutathione metabolism.^12^ Furthermore, vitamin C can directly reduce methemoglobin and is proven to treat cyanosis effectively.^13^ Vitamin E is an antioxidant, protecting the RBC from hemolysis induced through lipid peroxidation and the oxidation of sulfhydryl groups.^14^^,^^15^ Ascorbic acid and alpha-tocopherol in combination have been shown to have similar protecting effect on erythrocyte membranes exposed to an external oxidative stress.^16^^,^^17^ Vitamin B_1_ (thiamine) is known for its antioxidant properties. Thiamine interacts with free radicals and is oxidized to thiochrome and thiamine disulfide. The antioxidant effect of thiamine is probably related to successive transfer of 2H^+^ from the NH_2_ group of the pyrimidine ring and H^+^ from the thiazole ring.^18^ β-Carotene is a nontoxic precursor of vitamin A which scavenge free radicals to physically quench singlet oxygen reducing the extent of nuclear damage and inhibiting lipid peroxidation.^19^^, ^^20^

As far as we know there are no documented reports on the protective effect of vitamin C, E, A and B_1_ against oxidative stress in experimental models such as bovine erythrocytes. Therefore, the present study evaluates the antioxidative effects of particular vitamins on methemoglobinemia induced in* vitro* by sodium nitrite.

## Materials and Methods

Blood samples were collected from jugular vein of healthy dairy cattle and anticoagulated in EDTA. About 0.5 mL of each blood sample was exposed to three different concentrations (5, 10, 20 mmol L^-1^) of a vitamin (E, C, B1, A, and a combination of vitamin E and C) as antioxidant agent, separately. The selected concentrations were according to the previous study.^1^ All samples were incubated at 4 ^°^C for 24 hours. A control group was treated with normal saline, instead of vitamin. Then, all samples were treated with sodium nitrite (10 mmol L^-1^) as an oxidant agent for 10 minutes. Methemoglobin formation was determined as oxidative damage indicator. This method was applied to each sample 5 times.

Determination of methemoglobin was carried out according to Beutler *et al.* using modified method of Evelyn and Malloy.^21^^,^^22^ Whole blood (100 µL) was added to 10 mL phosphate buffer, 16 mmol L^-1^ at pH 6.6. The absorbance of solution was measured after 5 min, at 630 nm against distilled water as blank, using spectrophotometer (Jenway- 6100). Neutralized sodium cyanide (50 µL) was added to the cuvette and mixed, and a second reading was made at 630 nm, after 5 min. Then the lysate was diluted in phosphate buffer and 50 µL potassium ferricyanide (20%) and 50 µL sodium cyanide (10%) were added. The absorbance was measured at 540 nm against the reactant blank, to determine the total amount of hemoglobin in the sample. The methemoglobin concentration was expressed as a percentage of total hemoglobin.

The statistical significance of the experimental data was analyzed using the ANOVA and Tukey’s tests. A *P* value less than 0.05 was considered to be significant.

## Results

Table 1 illustrates the effect of pre-incubation with different concentrations of vitamins on the formation of methemoglobin induced by nitrate in bovine blood samples.

**Fig. 1:**
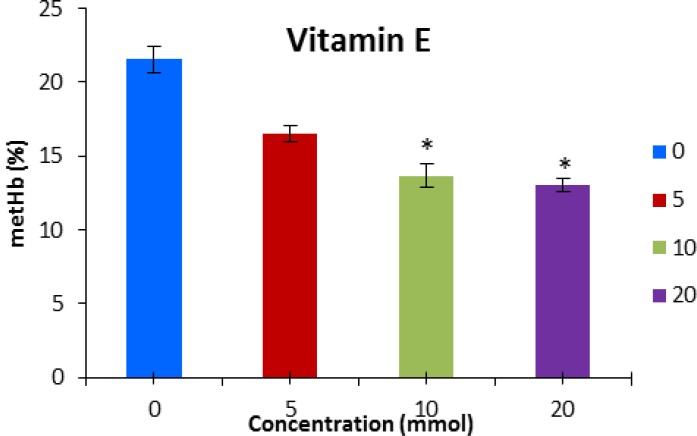
Methemoglobin concentration (mean ± SE) after pre-incubation with vitamin E. * indicates significant difference with control group (P < 0.05).

**Fig. 2:**
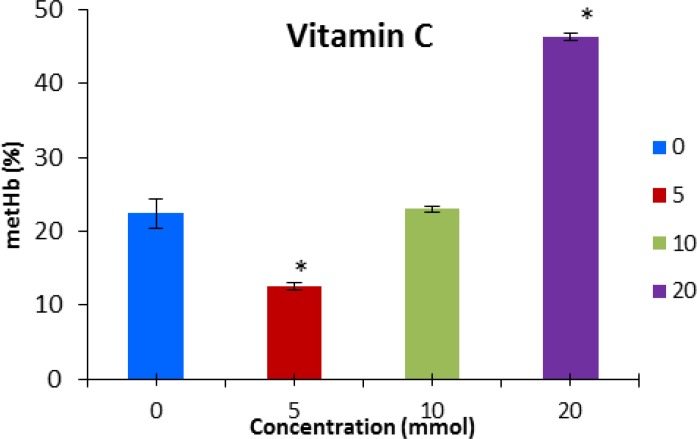
Methemoglobin concentration (mean ± SE) after pre-incubation with vitamin C. * indicates significant difference with control group (P < 0.05).

The results revealed that methemoglobin formation decreased significantly (*P* < 0.05) during pre-incubation with vitamin E at the concentration of 10 and 20 mmol L^-1 ^(Fig. 1). The level of methemoglobin was significantly lower than the control group when vitamin C (5 mmol L^-1^) was applied to the tests (Fig. 2).

**Table 1:** Effect of pre-incubation of particular vitamins with different concentrations on the formation of methemoglobin (as percentage mean ± SE) induced by nitrite in bovine blood samples

	Vitamin concentration (mmol L^-1^)			
	Control	5	10	20
Vitamin E	21.56 ± 0.87	16.51 ± 0.52	13.67 ± 0.79*	13.03 ± 0.46
Vitamin C	22.40 ± 2.02	12.57 ± 0.45*	22.98 ± 0.31	46.30 ± 0.57*
Vitamin B1	19.38 ± 0.95	20.08 ± 1.63	18.92 ± 2.54	20.05 ± 0.67
Vitamin E & C	20.58 ± 1.09	14.38 ± 0.79*	21.23 ± 0.97	25.53 ± 0.69*
Vitamin A	23.12 ± 1.07	22.97 ± 1.54	26.90 ± 1.72	28.13 ± 3.34

* indicates significant difference with the control group (*P* < 0.05).

**Fig. 3:**
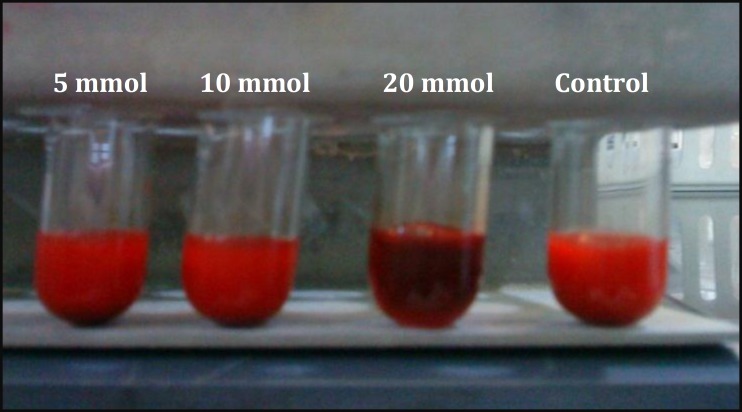
Blood samples appearance after pre-incubation with different concentrations of vitamin C.

Vitamin C, not only lost its antioxidant effect, but it also promoted an increase in methemoglobin formation at the concentration of 20 mmol L^-1^ (Fig. 3).

**Fig. 4:**
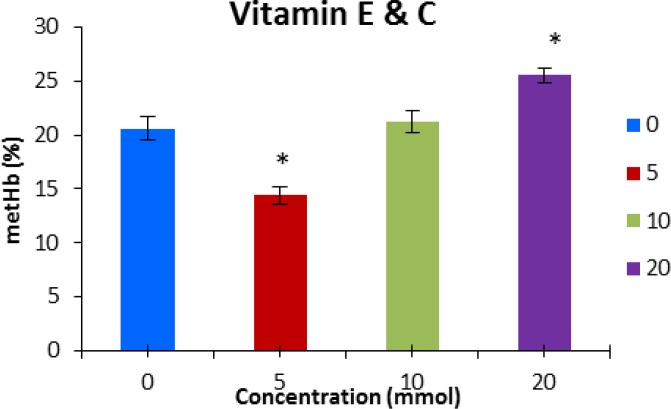
Methemoglobin concentration (mean ± SE) after pre-incubation with vitamin E and C. * indicates significant difference with control group (P < 0.05).

The combination of vitamins E and C, significantly decreased methemoglobin at the concentration of 5 mmol L^-1^, but increased it at 20 mmol L^-1^ (Fig. 4).

**Fig. 5:**
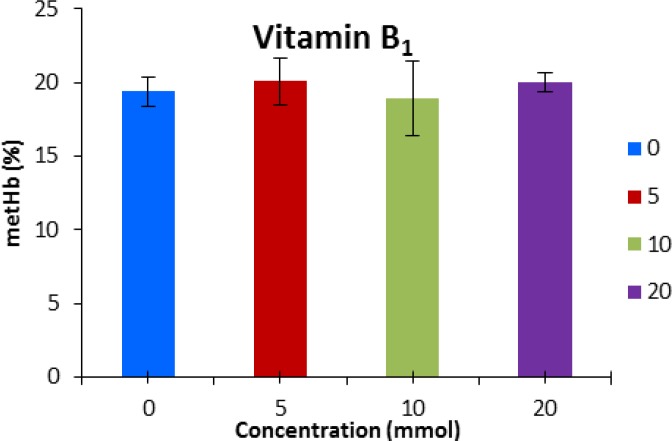
Methemoglobin concentration (mean ± SE) after pre-incubation with vitamin B1. No significant differences were detected among groups

**Fig. 6:**
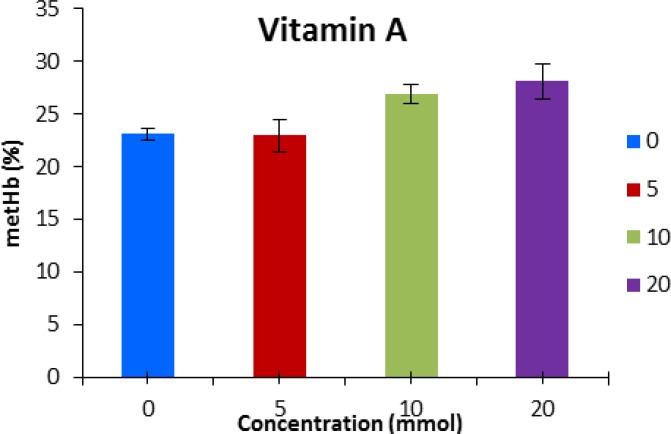
Methemoglobin concentration (mean ± SE) after pre-incubation with vitamin A. No significant differences were detected among groups

Vitamin A and B_1 _were not effective in decreasing methemoglobin at any concentration (Fig. 5 and 6).

## Discussion

Nitrite intoxication is a critical situation in ruminants and application of antioxidants such as vitamins may be useful in treatment. This study was performed to evaluate the antioxidative effects of some vitamins on methemoglobinemia which is induced *in vitro* by sodium nitrite.

The present study demonstrates that vitamin E at the concentration of 10 and 20 mmol L^-1^ decreased sodium nitrite induced methemoglobin formation. Hirneth and Classen showed that adding tocopherol or ascorbic acid to the diet could reduce formation of plasma nitrite and methemoglobin after oral treatment with NaNO_3_ in rats.^24^ Treatment with vitamin E significantly attenuates the elevation in the level of lipid peroxidation and hemoglobin autoxidation, and also this vitamin acts as an anti-oxidant on cadmium toxicity which the later, reduces superoxide dismutase, glutathione peroxidase and catalase activities and cause oxidative effect^25^ Vitamin E acts as an anti-oxidant, protecting RBCs from hemolysis induced by oxidative stress.^23^ It seems this vitamin counteracts with different species of oxidizers derived from H_2_O_2_ resulting in prevention of lipid peroxidation and the oxidation of sulfhydryl groups.^14^ Alhassan *et al.* suggested that vitamin E and combination of vitamin E and C are able to protect erythrocyte membrane alleviating the risk of increased heat-stress-induced hemolysis during hot dry season. ^26^

Our results showed that the application of vitamin C (5 and 10 mmol L^-1^) reduced the level of methemoglobin which is in agreement with other studies. Calabrese *et al.* explained that ascorbic acid significantly reduced the *in vitro* occurrence of sodium nitrite-induced methemoglobin (METHB) formation at a dose-dependent manner in erythrocytes from G6PD-deficient humans.^27^ Rajabi and Ale Davood suggested that treatment with ascorbic acid was possible in methemoglobinemic condition caused by toxic effects or by congenital methemoglobin reductase deficiency.^28^ However, methylene blue therapy is necessary when methemoglobin content of the blood is critically increased more than 30%. 

Vitamin C is recommended for treatment of methemoglobinemia. It has been demonstrated that the reduction of the methemoglobin formation occurs at low vitamin C concentration in mice erythrocytes.^29^ Claro *et al.* showed that Vitamin C (10–80 mmol L^-1^) prevents the formation of methemoglobin by phenylhydrazine, but did not have any effect on methemoglobin formation at the concentration of 90 mmol L^-1^.^30^

In our experiment, vitamin C at the concentration of 20 mmol L^-1 ^was not effective but it increased methemoglobin formation, significantly (*P *< 0.05). It might be related to the pro-oxidant effects of ascorbic acid at a particular concentration. Poljsak and Raspor reported that vitamin C causes oxidative damage in high level and in the presence of some metals such as iron, copper and chrome.^31^ similarly, ascorbic acid is known to reduce molecular oxygen to superoxide anion leading to the formation of hydrogen peroxide.^32^

Vitamin A and B_1 _did not seem to be efficient at any concentration which was used in this experiment. This was probably due to the short concentration and/or incubation period that was applied.

Beta-carotene supplementation can prevent ethanol-induced liver damage and increase glutathione concentrations in erythrocytes and the liver.^33^ Vitamin E and C combined with different doses of beta-carotene supplementation could improve ATPase activities and fluidity of erythrocyte membrane in the elderly people.^34^

Marouf *et al.* reported that benfotiamine, a lipid-soluble thiamine precursor, can effectively attenuate sodium nitrite-induced hemoglobin oxidation and maintain integrity of red blood cells, in concentration-dependent pattern, both *in vitro *and *in vivo*.^35^

During arsenic exposure, oral treatment with thiamin in rats did not result in significant decrease in erythrocytic lipid peroxidation and increase in catalase and superoxide dismutase activity.^36^

It was concluded that some vitamins especially vitamin C and E can reduce oxidative induced methemoglobin formation *in vitro* in a dose dependent manner, separately or in combination, and can be used as an alternative medication.
